# Polyphenol-rich cocoa in reducing diabetes-related pulmonary and vascular dysfunction: Protocol for a randomized controlled trial

**DOI:** 10.1016/j.ijcrp.2026.200706

**Published:** 2026-09-17

**Authors:** Lade Wosornu, Edward Amporful, Isabella A. Quakyi, Yaw Adu-Gyamfi, Frederick K. Addai, Francis Prince Ankrah, Jerry Owusu Ansah, Eric Fordjuor, Benjamin Larweh, Michael Owusu Manu, Martha Gyansa-Lutterodt, Joycelyn Azeez, Joseph Adjetey Oliver-Commey, Ebenezer Oduro-Mensah, Vincent Okyere Akomeah, Yao Mfodwo, Alfred Ampomah Appiah, Isaiah Agorinya, Timothy Adingiyuure Awine, Nana Agyenim Boateng, Yacoba Atiase, Charles Frederick Hayfron-Benjamin, George Obeng Adjei

**Affiliations:** a Ghana Academy of Arts and Sciences, Accra, Ghana; b Ghana Cocoa Board, Accra, Ghana; c Department of Biological, Environmental and Occupational Health Sciences, School of Public Health, University of Ghana, Ghana; d Department of Anatomy, University of Ghana Medical School, Accra, Ghana; e Ministry of Health, Accra, Ghana; f Ghana Infectious Diseases Centre, Accra, Ghana; g Ga East Municipal Hospital, Accra, Ghana; h Cocoa Marketing Co. Ltd, Accra, Ghana; i Mi-Cocoa Pty Ltd, Accra, Ghana; j Centre for Plant Medicine Research, Mampong-Akwapim, Ghana; k University of Health and Allied Sciences, Ho, Ghana; l Department of Medicine, University of Ghana Medical School, Accra, Ghana; m Departments of Internal Medicine, Vascular Medicine, and Respiratory Medicine, Amsterdam UMC, University of Amsterdam, the Netherlands; n Department of Physiology, University of Ghana Medical School, Accra, Ghana; o Department of Anaesthesia / Intensive Care, University of Ghana Medical Centre and Korle Bu Teaching Hospital, Accra, Ghana; p Centre for Tropical Clinical Pharmacology & Therapeutics, University of Ghana Medical School, Accra, Ghana

## Abstract

**Background:**

Pulmonary and vascular complications in diabetes remain highly prevalent and remain leading causes of morbidity and mortality, including sudden death. Recent studies suggest that therapies targeting low-grade inflammation and oxidative stress could halt the decline in pulmonary/vascular function in diabetes. One potential therapy is naturally occurring polyphenols. This study aims to investigate the potential pulmonary/vascular benefits of polyphenols in individuals with diabetes.

**Methods:**

This is a 2-arm, parallel, randomized controlled trial with 1:1 randomization, involving 100 participants per arm. Participants will receive either 500 mg of polyphenol (in 10 g of polyphenol-rich cocoa) per day for three months (intervention group) or an identical-appearing placebo once daily for three months, in addition to standard of care. Clinical, lifestyle, biochemical, pulmonary, microvascular, and macrovascular measures will be taken at baseline (week 0), and at weeks 4, 8, and 12 of treatment. The primary endpoint is the change in percentage predicted forced expiratory volume in 1 s (FEV_1_) from baseline to 12 weeks. Key secondary endpoints include changes in microvascular function and macrovascular function measures from baseline to 12 weeks. The changes in % predicted FEV_1_ and secondary endpoints over time will be analyzed using linear mixed-effects models with fixed effects for treatment group, time (baseline, 4, 8, and 12 weeks), and the treatment group–by–time interaction. Safety will be assessed descriptively, with the number (percentage) of participants experiencing (serious) adverse events summarized by treatment group.

**Conclusion:**

This study will provide data on how polyphenol-rich cocoa differentially impacts pulmonary and vascular functions in diabetes.

## 1 Introduction

In 2021, 10.5% of the world's adult population, representing over half a billion people, had diabetes mellitus; this prevalence is projected to rise to 12.2% by 2045 [1]. Diabetes-related vascular complications remain prevalent, affecting about 80% of individuals with diabetes [2] and accounting for over two-thirds of deaths in affected individuals [2]. Pulmonary complications in diabetes are as frequent as the vascular complications [3], and may be clinically relevant given the evidence that in the general population, low forced expiratory volume in 1 s (FEV_1_) or decline in FEV_1_ is associated with mortality, including sudden death [[4], [5], [6]].

To date, interventions and treatments targeting vascular and pulmonary complications, including controlling the conventional risk factors, have yielded limited results. For example, there are currently no established therapies that reliably reverse established diabetes-related neuropathy [7]. Existing data show that low-grade inflammation may be an important driver of diabetes-related complications [8], making it a process that can be targeted in halting vascular and pulmonary complications in diabetes. One potential candidate therapy is the use of naturally occurring polyphenols. Chronic hyperglycaemia in diabetes directly contributes to vascular and pulmonary complications by increasing mitochondrial reactive oxygen species (ROS) production and activating glucose-dependent pathways, including advanced glycation end-product formation, the polyol pathway, and protein kinase C signalling [9] These mechanisms intensify oxidative stress, decrease endothelial nitric oxide bioavailability, and promote endothelial dysfunction, leukocyte adhesion, and low-grade inflammation. Such processes are central to the development of microvascular injury, macrovascular disease, and impaired pulmonary function in diabetes. Polyphenol-rich cocoa may influence these hyperglycaemia-driven pathways via several mechanisms: enhancing endogenous antioxidant defences via Nrf2 activation, improving endothelial nitric oxide synthase activity and nitric oxide production, and inhibiting NF-κB-mediated inflammatory signalling [10,11]. Recent clinical evidence demonstrates modest reductions in inflammatory markers and improvements in endothelial function following flavanol-rich cocoa intake, supporting the hypothesis that polyphenols may reduce oxidative stress, vascular inflammation, and microvascular dysfunction in diabetes [12]^.^ These mechanistic findings offer a strong rationale for investigating polyphenols as a prospective strategy to improve vascular and pulmonary health in this study.

In a Cochrane review that included 35 trials, intake of flavanol-rich cocoa products was associated with a significant systolic and diastolic blood pressure-reducing effect [13]. While improving blood pressure may enhance vascular function in the setting of diabetes, polyphenols may also enhance vascular and pulmonary function via their antioxidant, anti-inflammatory, and anti-atherogenic activities [14]. Polyphenols may inhibit low-density lipoprotein (LDL) oxidation, lipoxygenase activity, inflammatory gene expression, and expression of genes encoding for cell adhesion proteins, chemotactic factors, and metalloproteinases [14]. Polyphenols may also increase endothelial nitric oxide synthase activity, inhibit arginase activity, inhibit platelet aggregation, and alter blood cholesterol concentrations, including an increase in HDL and a decrease in LDL and triglyceride concentrations [14]. These mechanisms have been demonstrated in in vitro and cell-culture studies, as well as animal studies performed with cocoa polyphenols [14].

The potential vascular and pulmonary benefits of cocoa polyphenols should also be considered in the broader context of metabolic and cardiometabolic dysfunction in diabetes. The triglyceride-glucose (TyG) index, a surrogate marker of insulin resistance, has been associated with long-term major adverse cardiovascular events in individuals at high cardiovascular risk and with mortality and appropriate implantable cardioverter-defibrillator therapy in patients with heart failure [15,16]. These findings underscore the contribution of insulin resistance and metabolic dysfunction to cardiovascular disease. Similarly, sodium-glucose cotransporter-2 (SGLT2) inhibitors have demonstrated cardiometabolic benefits in heart failure, including favorable effects on right ventricular function [17]. Collectively, these observations highlight the close interplay between metabolic dysfunction, vascular health, and cardiopulmonary function and provide a relevant context for investigating whether the antioxidant, anti-inflammatory, and endothelial effects of cocoa polyphenols can translate into improvements in vascular and pulmonary function in diabetes.

To the best of our knowledge, there are no reports of previous work exploring the impact of polyphenols on complementary measures of microvascular function. Secondly, the few studies that have explored the role of polyphenols in macrovascular health have focused mainly on coronary artery disease (CAD), and no study has explored the differential roles of polyphenols in improving peripheral artery disease (PAD) or cerebrovascular disease. A previous report has shown varying impacts of various vascular risk factors on atherogenesis in different vascular beds [18]. Finally, how polyphenol intake affects pulmonary function in diabetes is unknown. This proposed study seeks to answer these questions by evaluating the potential vascular and pulmonary benefits of polyphenols in a sub-Saharan African population.

## 2 Methods

### Ethical approval

The protocol is approved by the Institutional Review Board of the Korle Bu Teaching Hospital (Number: KBTH-IRB 000226/2023) and will be conducted in conformity with the Helsinki Declaration on Human Experimentation with the most recent revision (2024). The protocol is registered at the Pan African Clinical Trial Registry (PACTR202506573782241). No aspect of the protocol will be modified without the prior approval of the Institutional Review Board. Any protocol amendment will be effected in the trial registry and relevant stakeholders, including study participants, will be notified.

### 2.1 Patient and public involvement

The NDMRC had recently hosted a study evaluating pulmonary and vascular function in Ghanaian adults with diabetes. Participants from that study were asked to indicate their preferences regarding the study procedures. Their recommendations were factored into developing the protocol before submission to the Institutional Review Board. In the first week of the trial (pilot phase), in-depth interviews were conducted to explore patients’ preferences about the study procedures, and their recommendations were factored into developing the final treatment pathway.

### 2.2 Study design and setting

The proposed study is a prospective, randomized, double-blind, placebo-controlled, parallel-group clinical trial enrolling 200 participants with diabetes, who are equally allocated to standard of care alone or standard of care plus oral polyphenol-rich cocoa. Participants will be followed up at least once every month at weeks 4, 8, and 12 following the baseline visit and commencement of treatment. The study sites are the National Diabetes Management and Research Centre (NDMRC) in Accra, Ghana, and the Department of Physiology of the University of Ghana Medical School.

### 2.3 Study schedule

Patient recruitment for the study began on July 8, 2024, and we anticipate completing it by October 30, 2025. At baseline, participants will complete a survey and undergo biochemical, vascular, and pulmonary function assessments. Monthly follow-up visits will be scheduled for a period of three months during which the same assessments will be repeated (see Table 1). To reduce loss to follow-up, the final follow-up visit will align with participants' regular quarterly clinic visits. Additionally, participants will receive phone reminders and fasting instructions the day before each scheduled visit.

**Table 1:** Schedule of procedures.

Procedures	Visit				
	Prescreening	Baseline visit, Week 0	Follow-up visit #1, Week 4	Follow-up visit #2, Week 8	Follow-up visit #3, Week 12
Invitation to study and eligibility screening	X				
Informed consent		X			
Administration of intervention agent or placebo (30/31 packs)		X	X	X	
Demographics		X			
Completion of Clinical Abstraction Form		X	X	X	X
Lifestyle/behavioral characteristics (survey)		X	X	X	X
Cardiometabolic risk evaluation (survey)		X	X	X	X
Respiratory disease risk evaluation (survey)		X	X	X	X
Physical examination, including anthropometry		X	X	X	X
Biological sampling (including blood and urine) and biochemical/hematological testing		X	X	X	X
Respiratory function testing		X	X	X	X
Microvascular function testing		X	X	X	X
Cardiovascular/macrovascular function testing		X	X	X	X
Monthly evaluation of compliance with taking the intervention agent/placebo (via telephone calls)			X	X	X
Monthly evaluation of adverse events between visits (via telephone calls)			X	X	X
Study evaluation feedback surveys					X

### 2.4 Sample size

The sample size determination is based on the primary endpoint, defined as the difference or change in percentage predicted FEV_1_ from baseline to 12 weeks between the intervention and placebo group. Opinions on what constitutes a minimal clinically important difference (MCID) for FEV_1_ vary, and there is no internationally agreed consensus [19]. Regulators consider a % predicted FEV_1_ change of 5 to 10% from baseline as clinically important and a change of less than 3% from baseline as not clinically important [20]. Because the present study was conducted in individuals with diabetes rather than patients with primary pulmonary disease, we considered a smaller change to be potentially relevant to the more subtle pulmonary impairment associated with diabetes. Accordingly, a conservative minimum clinically relevant between-group difference of 4 percentage points in percentage-predicted FEV_1_ was assumed for the sample-size calculation. This value was selected to represent a difference that is greater than the range considered unlikely to be clinically important (<3%) but below the 5–10% threshold generally considered clinically important, thereby providing a conservative estimate appropriate for a population without primary lung disease. The standard deviation of change in % predicted FEV_1_ was assumed to be 12%, consistent with reported variability in longitudinal pulmonary function measures in adults [21]. Using a two-sided significance level of α = 0.05 and 80% statistical power, a total of 90 participants per group is required to detect this difference using a comparison of mean changes between groups, corresponding to a standardized effect size (Cohen's d) of approximately 0.33. Assuming an attrition rate of approximately 10%, the sample size comes to 100 participants per group.

### 2.5 Sampling procedure

A consecutive sampling procedure will be used, with the sample frame consisting of all patients with diabetes attending the NDMRC during routine clinic visits. All eligible patients who provide informed consent will be invited to participate in the study until the target sample size is reached. This approach aims to improve representativeness by systematically including all eligible patients throughout the recruitment period, reducing selection bias. A screening log will be completed by the principal investigator at the study site to document all study participants approached and reviewed for eligibility.

### 2.6 Participants

The study population includes Ghanaians aged ≥ 18 years with an established diagnosis of diabetes who are managed at the Diabetes Management and Research Centre in Accra, Ghana. The diagnosis of diabetes is based on fasting plasma glucose (FPG) ≥ 7.0 mmol/l and/or 2–h plasma glucose ≥ 11.1 mmol/l and/or glycated hemoglobin (HbA1c) of ≥6.5%, Pregnant women, individuals with known allergy to cocoa or polyphenols, and those with contraindications to spirometry [22] will be excluded from this study. Individuals included in the study will not take any cocoa product during the study period. To ensure this, all participants will be instructed to refrain from consuming cocoa-containing products during the study period. At enrollment, participants will receive standardized verbal and written guidance detailing common sources of cocoa. Compliance will be reinforced at each study visit through structured questioning and review of adherence checklists. To further support adherence, reminder messages will be provided during study visits via telephone calls. Any consumption of cocoa-containing products during the study will be documented as a protocol deviation, including the type of product, estimated amount consumed, and timing of intake. Participants reporting a single or minor deviation will be counselled to resume cocoa abstinence and will continue in the study. Repeated or substantial consumption will be classified as significant non-compliance and reviewed by the investigators to determine whether continued participation in the intervention is appropriate. All protocol deviations will be documented and retained in the study dataset and will be considered in pre-specified sensitivity analyses to assess their potential effect on the study findings. Informed consent will be obtained from participants by trained research assistants. Only individuals who give voluntary, competent, and appropriately informed consent will be recruited for the study.

### 2.7 Randomization

Participants will be randomized and allocated to either the intervention or control group. The Research Electronic Data Capture (REDCap) application will be used as the computerized randomization and data allocation system. This independent allocation method will be implemented to conceal the allocation sequence from the researcher and minimize the risk of bias. A double-blinding technique will be implemented for the participants and the researcher. Blinding supplies will be performed at a central pharmacy before distribution to investigators at the NDMRC. The double-blind would remain in place until all patients have completed their follow-up period.

### 2.8 Implementation and blinding

An independent research assistant not involved in recruitment, data collection, or analysis will prepare the study products. The intervention will comprise 10 g of natural cocoa powder containing 500 mg of polyphenol, while the placebo will comprise 1 g of brown food colorant without polyphenols. The 1-g quantity of food colorant was selected primarily to achieve a close visual match to the cocoa preparation. Both preparations will be dissolved in 100–200 mL of warm water before consumption. At this volume, 1 g of the food colorant produces a colour comparable to that of 10 g of natural cocoa prepared under the same conditions, whereas higher quantities result in a darker solution that does not closely resemble the cocoa preparation. Dissolving both preparations in the same volume of warm water also minimizes differences in mouthfeel associated with the powdered preparations. The intervention and placebo will be packaged in identical envelopes and provided with standardized preparation and consumption instructions.

Each dose will be pre-weighed using a Mettler Toledo scale, packaged in identical plastic dispensing envelopes, and placed in a secondary paper envelope bearing a unique study code generated by computer-based randomization. The bulk study products will be stored under controlled laboratory conditions at 18°C (±2°C) and relative humidity below 50%, in a dark, volatile-free environment prior to dispensing. The study products will be stored and distributed in identical envelopes according to the randomization sequence. The study products will be dispensed to participants at scheduled study visits according to the randomization sequence. Upon receiving their take-home supply, participants will be provided with standardized verbal and written instructions to maintain the study products in appropriate storage conditions, specifically in a cool, dry and dark location away from direct sunlight, moisture, and heat sources until consumption. The allocation key will be securely stored and remain inaccessible to investigators until completion of the data analysis. Study personnel who dispense the study products and the study participants will remain unaware of product identity, composition, or quantity. Participants will receive identical, scripted instructions and will not be informed of any differences between study products. Investigators, participants, outcome assessors, and data analysts will remain blinded throughout the study, with unblinding occurring only after database lock.

### 2.9 Intervention

The 200 study participants will be assigned to receive 500 mg of polyphenol in 10 g of polyphenol-rich cocoa per day for three months (in the active intervention group) or an identical-appearing placebo once daily for three months (in the control group), in addition to their usual clinical care by the physicians at the NDMRC.

For each of the three months, participants randomized to the intervention arm will receive 30 or 31 plastic dispensing envelopes, each containing 500 mg of polyphenol. Total phenolic content (TPC) of the cocoa powder was determined using the method described by Waterhouse. [23]. Briefly, 20 g of powdered cocoa was extracted with 25 mL of a methanol, distilled water, and acetone mixture (60:30:10, v/v/v) in a centrifuge tube. The mixture was allowed to stand at room temperature for 2 h and was subsequently centrifuged at 4000 rpm for 20 min using a Z326K centrifuge (Hermle Labortechnik, Wehingen, Germany). The supernatant was collected and stored at −20°C until analysis. TPC was determined using the Folin–Ciocalteu assay as described by Waterhouse [23]. Briefly, 50 μL of the crude extract was mixed with 125 μL of Folin–Ciocalteu reagent and 7.5% sodium carbonate solution. The mixture was incubated in the dark for 1 h, and absorbance was subsequently measured at 765 nm using a UV–visible spectrophotometer (TU-1810; Purkinje General Instrument Co., Ltd., Beijing, China). TPC was quantified using a calibration curve prepared with gallic acid and expressed as grams of gallic acid equivalents per gram of dry cocoa powder (g GAE/g dry powder). The Folin–Ciocalteu assay provides an estimate of total phenolic content and does not quantify individual polyphenolic compounds. The measured TPC was therefore used to characterize the overall phenolic content of the polyphenol-rich cocoa preparation used in the intervention.

The cocoa intervention was standardized to provide 10 g of cocoa powder containing 500 mg of polyphenol, as determined by laboratory quantification using the Folin–Ciocalteu method. All cocoa powder was obtained from a single manufacturer and production lot to minimize batch-to-batch variability and was subsequently portioned into individual 10-g doses using a standardized procedure under uniform storage and handling conditions. The same extraction and analytical procedures were applied to the cocoa samples throughout the study.

For each of the three months, participants randomized to the control arm will receive 30 or 31 plastic dispensing envelopes, each containing 1 g of food color.

At home, each participant will be instructed to add 100–200 ml of warm water to the intervention product (cocoa product or placebo), stir, and drink. Study participants will be seen at the clinic in weeks 4, 8, and 12 of treatment, at which time they will be queried about any side effects of the intervention and have the evaluation of microvascular, macrovascular, and pulmonary function, as well as measurement of diabetes, vascular, and pulmonary risk factors. A structured approach will be utilized to elicit adverse event (AE) occurrence, and participants will be encouraged to self-report details of any symptoms during the study period. All serious adverse event (SAE) reports will be submitted in a CIOMS-consistent format within 48 h of the site becoming aware of the event. Adverse effects will be assessed for causality, severity, and expectedness in accordance with ICH GCP principles (EU Directive 2001/20/EC Article 2).

Polyphenol-rich cocoa is already widely used, has an established safety profile, and no major unexpected adverse effects were anticipated. In addition, the trial duration is relatively short (3 months), and the study is conducted at a single site. Accordingly [24], the Institutional Review Board (IRB) of Korle Bu Teaching Hospital (KBTH) and the study principal investigator will monitor the trial. Given these, an independent Data Monitoring Board (DMB)/Data Safety Monitoring Board (DSMB) was not operationalized during the trial. The KBTH IRB will have the authority to request interim safety data, mandate protocol modifications, or suspend the study if safety concerns arise. The investigators will provide mandatory, real-time oversight of participants’ progress in accordance with the protocol, and the principal investigator will report all adverse events and protocol deviations to the KBTH IRB.

To enhance adherence to both the intervention and comparator protocols, participants will receive detailed counseling at enrollment on the importance of consistent medication use, supported by clear written instructions. Adherence will be reinforced through regular follow-up telephone calls or text messages before scheduled assessments. Study staff will perform an interview at each follow-up visit to assess compliance, and any barriers to adherence will be addressed promptly.

### 2.10 Variables and measurements

To maintain high data quality, all study staff were trained in data collection methods, including survey administration, physiological measurements, biological sample handling, and biochemical assessments, before the study started. Only trained and certified clinicians, technologists, or physiologists will be allowed to perform physiological and functional assessments. When applicable, anthropometric, hemodynamic, microvascular, macrovascular, and pulmonary function measurements will be obtained at least twice, in accordance with established guidelines.

At the baseline visit, a structured questionnaire will be used to capture the sociodemographic, lifestyle, and clinical characteristics of participants. A clinical abstraction form will be used to retrieve data on the medical history and therapy received by study participants. Further, structured questionnaires will be used to assess the respiratory and vascular function of study participants. Physical examinations, as well as biochemical, hematological, vascular, and pulmonary measurements, will be done at each of the study visits.

#### 2.10.1 Physical examination

A physical examination will be performed in accordance with the NDMRC's standardized operational procedures. The standing heights (without shoes) (to the nearest 0.1 cm) will be measured using the Seca 217 stadiometer. Body mass (to the nearest 0.1 kg) will be measured with study participants barefoot and in light clothing using the Seca 877 scale. Waist circumference will be measured in the fasting state with the Seca 201 girth circumference measuring tape at the midpoint between the lower margin of the last palpable rib and the iliac crest, and at the end of a normal expiration. Hip circumference will be measured with the Seca 201 girth circumference measuring tape at the widest point, measured at the level of the greater trochanters. The percentage of body fat and visceral body fat level will be measured using the arm-leg bio-impedance technique using the Omron Body Composition BF-506 Monitor. Blood pressure will be measured thrice using the Omron BP Monitor HEM-907XL with appropriate-sized cuffs after at least 5 min of rest while seated. The mean of the last two BP measurements will be used for the analysis.

#### 2.10.2 Biochemical and hematological analysis

The study participants will be adequately fasted (8-12 h) and have 10 ml of venous blood collected. Fasting plasma glucose, HbA1c, serum urea, creatinine, and lipids (triglycerides, total cholesterol, HDL-cholesterol) will be measured using the HumaStar 100 automatic biochemistry analyzer (HUMAN Diagnostic Worldwide, Germany). The estimated glomerular filtration rate (eGFR) will be calculated from the plasma creatinine levels using the race-neutral 2021 Chronic Kidney Disease Epidemiology Collaboration equation. Serum electrolyte concentrations will be measured with the Humanlyte electrolyte analyzer (HUMAN Diagnostic Worldwide, Germany) using ion-selective electrode technology. The enzyme-linked immunosorbent assay method will be used to detect anti-GAD, C-peptide, and human insulin. High-sensitivity CRP (hs-CRP) levels will be measured in heparin plasma by a particle-enhanced immunoturbidimetric assay. Human CRP agglutinates with latex particles will be coated with monoclonal anti-CRP antibodies. The aggregates will be determined turbidimetrically using the Pentra 400 chemistry analyzer (HORIBA ABX, Montpellier, France). Full blood count will be measured using the Humanlyte Analyzer by Sysmex. Total plasma polyphenol concentrations will be determined using the Folin–Ciocalteu colorimetric assay, with gallic acid as the calibration standard.

#### 2.10.3 Pulmonary function assessment

Peripheral capillary oxygen saturation (SpO_2_) and pulse rate will be measured with the WristOx2® Model 3150 (Nonin Medical, USA) using finger probes. Prebronchodilator spirometry in the sitting (previously described) [25] and supine positions will be conducted by trained physicians/technicians using the Vitalograph Pneumotrac Portable Screening Pneumotachograph (Vitalograph Ltd) according to the American Thoracic Society/European Respiratory Society (ATS/ERS) guidelines [26]. Prebronchodilator spirometry in the sitting and supine positions will be performed using the same technique, except for the position the patient assumes (i.e., sitting or supine). The predicted values of FEV_1_, FVC, and FEV_1_/FVC will be determined for each participant using the Global Lung Function Initiative 2012 equations using both race-neutral and race-specific average reference equations. The values of FEV_1_/FVC and FVC will be used to categorize pulmonary function patterns as normal, obstructive, restrictive, or mixed obstructive and restrictive based on ATS/ERS guidelines [27]. For study participants with obstructive airway patterns based on the sitting prebronchodilator spirometry, postbronchodilator spirometry will be performed 15 min after administration of 200 μg salbutamol via metered-dose inhaler with spacer. The change in FVC from the sitting to supine position will be determined and expressed as a percentage of the sitting FVC. Diaphragm muscle weakness will be defined **as** a supine FVC≥10% lower than a sitting FVC [28].

#### 2.10.4 Microvascular dysfunction assessment

Vibration perception threshold (VPT) will be measured using the Horwell neurothesiometer (Scientific Laboratory Supplies Ltd, Nottingham) at the metatarsophalangeal joint of both feet in a two-step manner (ascending and descending methods); this has been previously described [3]. The arithmetic means of the measured ascending and descending VPTs will be performed, and the arithmetic means of consistent values will be used as the final descending VPT used in the analyses. Symptoms of diabetic neuropathy will be scored with the Diabetic Neuropathy Symptom (DNS) score [29]. Neuropathy will be diagnosed if the VPT is ≥ 25V [30] and/or a DNS score is ≥ 1 ^29^.

The ZEISS 500 Fundus Camera (ZEISS Inc. JENA) will be used for retinal photography after dilatation with tropicamide (1%) and phenylephrine (2.5%) ophthalmic solutions; this has been previously described [3]. Retinal images will be analyzed and graded by a certified ophthalmologist according to the Early Treatment Diabetic Retinopathy Study (ETDRS) criteria [31]. Diabetic retinopathy, when present, will be classified as either proliferative diabetic retinopathy (PDR) or non-proliferative diabetic retinopathy (NPDR), and NPDR will be graded as mild, moderate, severe, or very severe. Diabetic macular edema will be considered present when there is some apparent retinal thickening or hard exudates in the posterior pole.

Direct analyses of urinary albumin and creatinine concentrations will be performed on an early-morning urine sample. Urinary albumin concentration will be measured by an immunochemical turbidimetric method (Roche Diagnostics). Urinary creatinine concentration will be measured by a kinetic spectrophotometric method (Roche Diagnostics). The urinary albumin-creatinine ratio (ACR) will be calculated. Albuminuria classifications will be derived from the ACR as follows: A1, <3 mg/mmol (normal to mildly increased); A2, 3–30 mg/mmol (moderately increased), and A3, >30 mg/mmol (severely increased) [32]. Nephropathy based on albuminuria will be defined as ACR ≥ 3 mg/mmol (category ≥ A2) according to the 2012 Kidney Disease Improving Global Outcomes guideline [32].

#### 2.10.5 Macrovascular dysfunction assessment

Ankle-brachial pressure index (ABI) measurements will be performed in the supine position. Both the oscillometric and Doppler techniques will be used. The oscillometric technique has been previously described [33]. For the Doppler technique, ABI will be measured using the Huntleigh Doppler equipment with an 8 MHz Doppler probe (Huntleigh, UK). The Huntleigh Doppler equipment will be used to determine flow reappearance during the slow deflation of a blood pressure cuff. The systolic pressure of the posterior tibial and dorsalis pedis will be measured in both ankles, and the lower of the two will be used as the ABI numerator. The denominator will be determined by the higher of the 2 systolic blood pressure readings taken from each arm. PAD will be defined as ABI≤0.90 [34].

A standard 12-lead supine resting digital electrocardiogram (ECG) will be recorded using the Vitalograph BT12 ECG™ combined with its Spirotrac® software (Vitalograph, United Kingdom). Transmission of the ECG trace will be wireless with no leads to trip over, providing excellent signal quality, fewer artifacts, and ensuring minimal patient discomfort. The ECG tracings will be processed with the device's inbuilt University of Glasgow 12-Lead ECG Analysis Algorithm [35]. Two additional methods were used to evaluate each ECG, namely Minnesota coding and assessment by a trained physician. Symptoms of CAD will be assessed using the WHO Rose Angina Questionnaire [36]. CAD will be defined as 1) the presence of possible myocardial infarction or angina pectoris based on the Rose Angina Questionnaire and/or 2) the presence of pathological Q waves and/or ST segment changes in at least 2 contiguous leads on an electrocardiogram.

Stroke will be self-reported and confirmed by imaging methods such as head computed tomography scan or Magnetic Resonance Imaging.

### 2.11 Data analysis plan

Data collected will be entered into a locally built database hosted by the University of Ghana Medical School. Entries will be verified through a double-entry process. Appropriate file security measures will be implemented to maintain confidentiality. To minimize missing data, we will 1) obtain accurate participant contact information (and update it after each follow-up visit) and send visit reminders; 2) ensure double-entry within 10 days of data collection or availability; 3) Conduct weekly meetings with the study team to review progress and resolve any data management-related questions; 4) Perform biweekly random cross-checks of electronic data against source documents; and 5) Implement quality control measures, including data validation checks, to identify incomplete or inconsistent entries.

Study participants may exit the study due to reasons such as withdrawing consent, being lost to follow-up, or AE/SAE developed due to the intervention or placebo. Data collected from study participants who discontinue will be retained for analysis unless they specifically request removal, with the date and reason for discontinuation recorded. Missed visits will be flagged, and no data imputation will occur post-exit. Exiting participants will not be replaced, but their data may be used in longitudinal analyses. If high exit rates lead to recruitment shortfalls, additional participants may be enrolled to ensure sufficient statistical power. Missing data will be explored for patterns and reasons. Mixed-effects models inherently accommodate missing data under a missing-at-random assumption. If more than 5% of data are missing for key endpoints, multiple imputations will be performed as a sensitivity analysis. Additional sensitivity analyses will include per-protocol analyses and assessment of alternative covariance structures.

While randomization is expected to minimize baseline confounding, adjustment for a limited number of pre-specified baseline variables will be undertaken to improve the precision of the treatment-effect estimates. Potential covariates will be identified a priori using directed acyclic graphs (DAGs) for each exposure–outcome pair, together with consideration of their clinical and prognostic relevance. The primary model will be restricted to a small number of pre-specified covariates to minimize the risk of overfitting. For the primary pulmonary outcome, these will include baseline percentage-predicted FEV_1_, duration of diabetes, age, and sex. Other potential covariates, including smoking status, body mass index, physical activity, baseline measures of microvascular or macrovascular function, medication use, dietary supplement use, sodium intake, and alcohol consumption, will not be included simultaneously in the primary model but may be examined in pre-specified secondary or sensitivity analyses where clinically justified.

The potential confounders will include participant-related factors (e.g. age, sex, duration of diabetes, smoking, body mass index and its change, physical activity and baseline measures of pulmonary, microvascular or macrovascular function, medication related factors (anti-diabetic medications, antihypertensive drugs, lipid-lowering therapy, and respiratory medications), and dietary-related confounders (use of dietary supplements, sodium intake, and alcohol consumption),

All analyses will follow the intention-to-treat (ITT) principle, including all randomized participants according to their assigned groups. A per-protocol (PP) analysis will be conducted as a sensitivity analysis restricted to participants who adhered to the protocol. Safety analyses will include all participants who received at least one dose of the allocated intervention. Simple tables, proportions, means, and medians will be used to examine the data. Both graphical methods (including histograms and Q-Q plots) and formal statistical tests (including the Kolmogorov-Smirnov and Shapiro-Wilk tests) will be used to assess the normality of data. Data with a normal distribution will be presented as mean (standard deviation), whereas those not normally distributed will be presented as median (interquartile range). Categorical data will be presented as frequency (percentage). For continuous variables, mean and median values in two independent groups will be compared using a two-sample independent sample *t*-test (unpaired *t*-test) and the Mann-Whitney *U* test (Wilcoxon rank-sum test) for normally and not normally distributed variables, respectively.

The primary endpoint is the change in pulmonary function, assessed by % predicted FEV_1_, from baseline to 12 weeks. Key secondary endpoints include 1) changes in microvascular function from baseline to 12 weeks, assessed using urinary albumin to creatinine ratio and vibration perception threshold, and 2) changes in macrovascular function from baseline to 12 weeks, assessed using ankle brachial pressure index and maximum ST-segment depression measured at 60 ms after the J-point in ≥2 contiguous leads. The changes in % predicted FEV_1_ and secondary endpoints will be analyzed using linear mixed-effects models with fixed effects for treatment group, time (baseline, 4, 8, and 12 weeks), and the treatment group–by–time interaction. The primary endpoint will be tested at a two-sided significance level of α = 0.05. To control for multiple comparisons among secondary endpoints, the Holm–Bonferroni procedure will be applied. Results for secondary endpoints will be considered supportive of the primary analysis. While the pulmonary and vascular systems are distinct, alterations in these systems are related both mechanistically and in the clinical presentations in individuals with diabetes and the general population [37,38].

In addition to the primary and secondary physiological endpoints, the study will compare the occurrence of selected clinical events between the two groups during the 12-week follow-up period. These will include: 1) all-cause mortality; 2) major adverse cardiovascular events, defined as a composite of cardiovascular death, non-fatal myocardial infarction, or non-fatal stroke; 3) hospitalization for cardiometabolic causes (e.g., acute coronary syndrome, heart failure, arrhythmia, acute or chronic kidney disease, or acute diabetic complications); 4) hospitalization due to respiratory causes including respiratory failure; and 5) new onset or resolution of pre-existing pulmonary dysfunction, microvascular dysfunction, or macrovascular dysfunction. The incidence of these clinical events will be summarized by treatment group using counts (proportions). Between-group comparisons will be conducted using chi-square tests or Fisher's exact tests, as appropriate. Time-to-event analyses will be performed where feasible using Kaplan–Meier survival curves and compared between groups using the log-rank test. If event numbers permit, Cox proportional hazards models will be used to estimate hazard ratios with corresponding 95% confidence intervals, adjusting for relevant baseline covariates. These clinical event analyses are exploratory and are not powered to detect treatment differences in clinical event outcomes. Accordingly, the results will be interpreted primarily descriptively and as supportive evidence regarding the potential broader clinical effects of the intervention rather than as definitive evidence of efficacy. All tests will be two-sided with a significance level of 0.05. Analyses will be conducted using R version 4.6.1 (R Foundation for Statistical Computing, Vienna, Austria) or IBM SPSS Statistics version 32.0.0 (IBM Corp., Armonk, NY, USA).

### 2.12 Limitations

The trial has some limitations. First, it does not include individuals of other ethnic origins, including those of European or Asian ancestry. Second, although considerable effort was made to visually match the intervention and placebo, the placebo contained a smaller total powder mass than the intervention. Both preparations were dissolved in the same volume of warm water, and the placebo quantity was selected to achieve a comparable colour while minimizing differences in mouthfeel. Nevertheless, residual differences may have been present and could potentially have resulted in some degree of unblinding. Although blinding was not formally assessed, the use of identical packaging and standardized preparation and administration procedures, together with blinding of participants and study personnel to group allocation, was intended to minimize the likelihood of treatment allocation being inferred. However, the possibility of some degree of unblinding and its potential effects on performance or detection bias cannot be completely excluded.

### 2.13 Dissemination/publication plan

In addition to communicating the study results to participants, patient groups (especially those with diabetes), the NDMRC, the ethical review boards, and other relevant stakeholders in the Ghana healthcare system, the study results will be presented at relevant national and international scientific meetings and conferences and will be published in peer-reviewed journals.

## CRediT authorship contribution statement

**Lade Wosornu:** Writing – review & editing, Writing – original draft, Project administration, Methodology, Investigation, Funding acquisition, Conceptualization. **Edward Amporful:** Writing – review & editing, Writing – original draft, Methodology, Investigation, Funding acquisition, Conceptualization. **Isabella A. Quakyi:** Writing – review & editing, Writing – original draft, Methodology, Funding acquisition, Conceptualization. **Yaw Adu-Gyamfi:** Writing – review & editing, Writing – original draft, Methodology, Funding acquisition, Conceptualization. **Frederick K. Addai:** Writing – review & editing, Writing – original draft, Methodology, Funding acquisition, Conceptualization. **Francis Prince Ankrah:** Writing – review & editing, Writing – original draft, Funding acquisition, Conceptualization. **Jerry Owusu Ansah:** Writing – review & editing, Writing – original draft, Methodology, Conceptualization. **Eric Fordjuor:** Writing – review & editing, Writing – original draft, Methodology, Conceptualization. **Benjamin Larweh:** Writing – review & editing, Writing – original draft. **Michael Owusu Manu:** Writing – review & editing, Writing – original draft. **Martha Gyansa-Lutterodt:** Writing – review & editing, Writing – original draft, Methodology, Funding acquisition, Conceptualization. **Joycelyn Azeez:** Writing – review & editing, Writing – original draft, Conceptualization. **Joseph Adjetey Oliver-Commey:** Writing – review & editing, Writing – original draft, Conceptualization. **Ebenezer Oduro-Mensah:** Writing – review & editing, Writing – original draft. **Vincent Okyere Akomeah:** Writing – review & editing, Writing – original draft. **Yao Mfodwo:** Writing – review & editing, Writing – original draft. **Alfred Ampomah Appiah:** Writing – review & editing, Writing – original draft. **Isaiah Agorinya:** Writing – review & editing, Writing – original draft. **Timothy Adingiyuure Awine:** Writing – review & editing, Writing – original draft. **Nana Agyenim Boateng:** Writing – review & editing, Writing – original draft. **Yacoba Atiase:** Writing – review & editing, Writing – original draft. **Charles Frederick Hayfron-Benjamin:** Writing – review & editing, Writing – original draft, Project administration, Methodology, Investigation, Data curation, Conceptualization. **George Obeng Adjei:** Writing – review & editing, Writing – original draft, Project administration, Methodology, Investigation, Funding acquisition, Conceptualization.

## Data sharing

Data from this study may be provided to qualified researchers upon a reasonable request made to the study group. Data shared will be coded, with no protected health information included. Approval of the request and execution of all applicable agreements are prerequisites to the sharing of data with the requesting party.

## Funding statement

This study is funded by the Ghana Cocoa Board (COCOBOD). COCOBOD has no role in the study's design, execution, data analysis, interpretation, or decision to submit results. The website of COCOBOD is https://cocobod.gh/

Author Charles Frederick Hayfron-Benjamin was supported by the 10.13039/100000061Fogarty International Center and 10.13039/100000025National Institute of Mental Health and 10.13039/100000062National Institute of Diabetes and Digestive and Kidney Diseases of the 10.13039/100000002National Institutes of Health under Award Number D43TW010543. The content is solely the responsibility of the authors and does not necessarily represent the official views of the National Institutes of Health.

## Declaration of competing interests

Investigators of this research have no conflict of interest to declare.
